# Pre-Existing Heterogeneity Predicts Rare Proteostasis-Stress Programs Across Diverse Perturbations

**DOI:** 10.3390/biology15151230

**Published:** 2026-07-23

**Authors:** Zongnan Lyu, Chunxue Shao, Renyu Yang, Qi Yu, Guang Yang, Ziheng Wang

**Affiliations:** Division of Computational Biology, Chinese Center of Exercise Epidemiology, Northeast Normal University, Changchun 130024, China

**Keywords:** single-cell perturbation, proteostasis stress, rare high-stress populations, integrated stress response, unfolded protein response, baseline heterogeneity, stress-program prediction, heat shock response, predictability across perturbations

## Abstract

Stress-associated transcriptional programs are common in single-cell perturbation data, but they are often treated as technical or experimental nuisance signals. Whether rare high-stress populations arise stochastically after perturbation or reflect outcomes associated with pre-existing cellular heterogeneity remains unclear. Herein, we build a cross-dataset stress-program prediction framework spanning 146,321 single cells and 926 perturbation–cell-line tasks. Untreated baseline heterogeneity, together with perturbation identity, predicted future rare integrated-stress burden across held-out cell-line–drug pairs ($R^{2}=0.742$, Pearson r=0.862). Single-cell stress-program prediction generalized across leave-task-out, leave-cell-line-out and leave-perturbation-out splits; retained signal in leave-dataset-out evaluation; and collapsed to near-null performance under within-task label permutation. Independent validation datasets provided external support for the inferred stress axes: tunicamycin and thapsigargin activated unfolded protein response/integrated stress response (UPR/ISR) modules in bulk RNA sequencing (RNA-seq), thapsigargin expanded populations with high X-box binding protein 1 (*XBP1*) UPR or activating transcription factor 4 (*ATF4*) ISR activity in donor-paired single-cell data, and an independent protocol-stress dataset indicated heat shock/proteostasis structure beyond cell type and quality control (QC). The resulting resource summarizes perturbation responses as activator protein 1 (AP1) immediate, UPR/*ATF4*, heat shock, replication-coupled and low/mixed dominant stress-program patterns. These findings suggest that stress variation should not be viewed solely as a nuisance covariate but may represent a predictable and biologically structured dimension of perturbation response space.

## Simple Summary

Cells exposed to the same treatment do not always respond in the same way. A small subset can show strong stress activity linked to protein quality control, the cellular process that keeps proteins properly balanced and functional, but these signals are often dismissed as experimental noise. This study asked whether such stress responses appear randomly or can be anticipated from differences that already exist before treatment. We analyzed public gene activity measurements from individual cells across many laboratory-grown cell populations and treatments, then tested whether untreated cell populations and treatment identity could predict the later emergence of cells with high stress activity. The results showed that pre-existing cellular differences and treatment context were informative, that the stress responses reflected several related programs rather than one generic stress signal, and that independent datasets supported the main stress patterns. These findings suggest that stress variation in cell studies can contain useful biological information. This work may provide a basis for designing experiments, interpreting treatment responses more carefully, and identifying cellular contexts in which rare stress-prone cells are more likely to emerge.

## 1. Introduction

Single-cell perturbation profiling has indicated that cellular responses to the same stimulus are often heterogeneous rather than uniform [1,2,3,4,5]. Even genetically similar cells exposed to identical drugs, cytokines, or environmental challenges can occupy distinct transcriptional states, with only a subset entering extreme response programs [6,7,8]. Such rare transcriptional programs are of particular interest because they have been associated with treatment survival, failure of homeostatic recovery, and the emergence of context-specific vulnerabilities [8,9,10,11]. Among these heterogeneous responses, proteostasis-related programs—including the unfolded protein response (UPR), integrated stress response (ISR), heat shock signaling, activator protein 1 (AP1) immediate early activation, and cell-cycle stress pathways—represent key adaptive and damage-associated regulatory axes [12,13,14,15,16].

Despite their biological relevance, stress-associated transcriptional programs in single-cell data are difficult to interpret. These signals may reflect genuine adaptive responses to perturbation, but they may also arise from dissociation procedures, technical artifacts, or low-quality cells [17,18]. As a result, stress-related variation is frequently treated as a confounding factor and removed or regressed out in downstream analyses. While this improves certain analytical outcomes, it also raises the possibility that structured biological information may be inadvertently discarded. This leads to a fundamental question: whether rare high-stress populations represent stochastic and largely unpredictable outliers or whether their emergence is partially determined by pre-existing cellular heterogeneity in a context-dependent manner.

This question is particularly important because population-level summaries can obscure rare subpopulations that disproportionately influence biological outcomes [8,9,19]. Perturbations may induce only modest shifts in average expression while causing small but reproducible subsets of cells to exhibit high activity of the UPR, ISR, heat shock, or AP1 programs. Conversely, perturbations with comparable global stress burdens may engage distinct regulatory programs across different cellular contexts. Disentangling these possibilities requires analytical frameworks that can evaluate the predictability of rare stress-program emergence, assess generalization across independent datasets, and distinguish biological structure from technical and compositional confounding.

Previous work has established that single-cell perturbation profiling can resolve heterogeneous transcriptional responses to genetic and chemical perturbations that are obscured by population-average assays [1,2,5,20]. Computational studies have further shown that single-cell perturbation responses can be predicted or transported across conditions, supporting hold-out prediction as a way to test whether response structure generalizes beyond the training context [20,21]. Studies of drug tolerance and lineage/state-to-fate relationships further indicate that rare or pre-existing transcriptional states can be associated with later cellular outcomes [8,9,19,22]. In parallel, UPR/ISR, heat shock and proteostasis pathways have been characterized as major stress-response systems, and dissociation and tissue-processing studies have shown that stress signatures can also arise from protocol-associated effects [12,13,14,17,23]. However, it remains unclear whether rare populations with high proteostasis-stress activity observed across perturbation datasets are predictable outcomes associated with baseline heterogeneity or whether they are largely stochastic, context-specific or technical events.

Here, “rare” is used to describe cells occupying the high-activity tail of stress-module variation rather than a group defined by a fixed cell proportion, and stress axes are considered continuous and partially overlapping dimensions rather than discrete mutually exclusive categories; dominant stress-program pattern labels are therefore used only as summaries of relative module activity [19]. Using this conceptual framework, we aimed to determine whether pre-existing cellular heterogeneity, together with perturbation context, can anticipate rare proteostasis-stress emergence; whether stress-associated variation can separate overlapping UPR/ISR, heat shock, AP1 immediate early and replication-coupled axes from a single generic stress signal; and whether the inferred axes generalize across held-out perturbation contexts and remain consistent in independent validation datasets.

## 2. Materials and Methods

### 2.1. Study Design

This study was organized as a retrospective cross-dataset analysis designed to test whether rare proteostasis-stress programs are predictable and whether the inferred stress axes are reproducible across independent perturbation contexts. The workflow contained two linked analytical tasks. First, a baseline-to-future prediction task asked whether heterogeneity in untreated cell populations, combined with perturbation identity, could predict the future burden of rare high-integrated-stress populations after treatment. Second, a single-cell stress-program classification task asked whether cells in rare high-stress populations could be identified from interpretable stress, regulatory and quality-control features. These two tasks were followed by external validation analyses to assess whether the stress axes recovered from the discovery compendium were consistent with independent endoplasmic reticulum (ER) stress and protocol-stress datasets.

The discovery analyses used processed public single-cell perturbation resources and associated summary tables [3,4]. For each perturbation–cell-line task, untreated baseline summaries, per-cell module-score annotations and task-level perturbation summaries were analyzed in parallel. The study was observational and computational; therefore, all predictive analyses were interpreted as tests of generalizable association rather than as evidence of causal determination of future entry into a rare high-stress category.

### 2.2. Data Sources

The discovery compendium contained 146,321 single cells spanning 926 perturbation–cell-line tasks and 172 cell lines. A task was defined as a unique dataset, perturbation and cell-line context with corresponding single-cell module-score and metadata annotations. Baseline-to-future prediction used cell-line–drug-response summaries from the McFarland perturbation resource to relate untreated population heterogeneity to the rare post-treatment high-integrated-stress fraction [4]. Single-cell stress-program classification and stress-program resource construction used the per-cell prediction table and task-level perturbation summaries from the discovery compendium.

Three independent validation datasets were used to evaluate the external consistency of the inferred stress axes. GSE129757 was used as a bulk RNA sequencing (RNA-seq) ER stress validation dataset in LN308 cells treated with tunicamycin or thapsigargin for 2 or 6 h [24]. GSE251912 was used as a single-cell ER stress validation dataset, comprising human islet single-cell RNA sequencing (scRNA-seq) after 24 h dimethyl sulfoxide (DMSO) or thapsigargin exposure across three donors [25]. GSE140819 was used as a protocol-stress validation dataset, focusing on the non-small-cell lung cancer sample 14 (NSCLC14) matched-dissociation-protocol subset to assess heat shock and proteostasis structure beyond cell type and quality-control (QC) covariates [26].

### 2.3. Definitions of Stress Modules and Rare High-Stress Populations

Stress modules were defined from curated gene-set annotations representing major stress-response programs. Here, stress axes refer to continuous and potentially overlapping module-score dimensions summarizing coordinated stress-response programs, rather than discrete or mutually exclusive discrete cell categories. The fixed module panel included integrated stress, unfolded protein response/integrated stress response (UPR/ISR)-related modules, X-box binding protein 1 (*XBP1*) unfolded protein response, activating transcription factor 4 (*ATF4*) integrated stress, heat shock factor 1 (*HSF1*) heat shock, activator protein 1 (AP1) immediate early activation, E2F transcription-factor replication, tumor protein p53 (*TP53*) axis and nuclear factor κB (NF-κB) inflammatory stress modules where available [27,28,29,30,31,32,33]. Module definitions were kept constant across discovery and validation analyses so that differences among datasets reflected changes in module activity rather than changes in gene-set composition.

For single-cell data, module scores were computed after library-size normalization to 10,000 counts per cell, log(1+x) transformation, genewise z-score standardization across cells and averaging across detected genes within each module, consistent with common single-cell preprocessing and gene-signature scoring workflows [34,35]. For bulk RNA-seq validation, counts were first expressed as counts per million (CPM) and then converted to $\log_{2}\left(\mathrm{CPM}+1\right)$, z-scored genewise across samples and averaged across detected module genes. Module scores were interpreted as relative within-dataset activity estimates and were not treated as directly comparable absolute expression values across datasets.

Rare high-stress cells were defined by pre-specified module-score thresholds rather than by a fixed population-frequency cutoff. In the primary discovery and resource analyses, rare high-integrated-stress cells were defined as cells with integrated_stress_z >2, matching the binary target used for single-cell prediction and the task-level rare-fraction summaries. In the external single-cell validation analyses, high-module cells were summarized using the threshold z>1, matching the validation analyses in GSE251912 and GSE140819. Rare-state fractions were calculated as the proportion of cells exceeding the relevant threshold within each perturbation–cell-line task, donor–condition group, or protocol–cell-type stratum. Thus, “rare” refers to high-tail module-score membership under the specified analysis threshold, not to a fixed 1% or 5% frequency imposed across all datasets.

### 2.4. Modeling Framework

The baseline-to-future prediction model used ridge regression to predict the post-treatment rare integrated-stress fraction from untreated baseline features and perturbation identity. Predictor variables included baseline module-score means, standard deviations, upper quantiles and rare high-stress fractions, together with drug-indicator variables. Missing or infinite predictor values were imputed using the corresponding feature median and were set to zero if no finite median was available. Predictors were standardized, ridge regression was fitted with α=3.0, and generalization was evaluated using GroupKFold cross-validation grouped by cell line with up to five folds. Performance was summarized using hold-out $R^{2}$, Pearson correlation, Spearman correlation, mean absolute error and calibration estimates.

The single-cell stress-program classification model used the primary rare high-stress label defined above and compared logistic-regression and random-forest classifiers. Feature sets were designed to separate QC-only information from biological stress and regulatory information. QC-only features included ncounts and ngenes; replication/DNA damage response (DDR) features included DDR/p53 repair, cell-cycle replication, *TP53* and E2F modules; stress-module features included AP1 immediate, *ATF4* integrated, *HSF1* heat shock, *XBP1* UPR and NF-κB inflammatory stress; and upstream/non-direct feature sets excluded the direct integrated-stress target score. Logistic regression used standardized predictors, balanced class weights, C=0.5 and a maximum of 2000 iterations. Random forests used balanced subsample class weights, 220 trees and a minimum leaf size of 12; feature importance was estimated with an upstream/non-direct stress feature set using 350 trees. Models were implemented in scikit-learn and evaluated only on held-out folds [36,37].

The perturbation–cell-line stress-program resource was constructed by grouping cells by dataset, perturbation, cell line and task name. For each group, cell number, rare integrated-stress fraction, mean QC metrics, mean biological module scores and rare z>2 high-module fractions were recorded. No additional prevalence cutoff, such as requiring the rare fraction to be below a fixed percentage, was imposed during resource construction. Dominant stress-program patterns were assigned by comparing *XBP1*/*ATF4*, *HSF1* heat shock, AP1 immediate and E2F replication mean scores; tasks with maximum axis score below 0.5 were labeled low/mixed. This pattern assignment was used as a descriptive resource annotation rather than as a supervised training label.

### 2.5. Confounding Control

Confounding control focused on QC features, QC-residualized biological features, empirical label permutation and grouped hold-out evaluation. The QC features were ncounts and ngenes in the discovery compendium as well as unique molecular identifier count (nUMI), detected-gene count (nGene) and mitochondrial read fraction (percent_mito) where available in external single-cell datasets. For the biology-residualized QC configuration, each biological feature was residualized against QC variables using linear models fitted within the training fold; the training-fold coefficients were then applied to both training and held-out cells to avoid information leakage [38].

Single-cell generalization was evaluated using leave-task-out, leave-dataset-out, leave-cell-line-out and leave-perturbation-out grouped cross-validation. Groups with fewer than 20 cells were excluded from the corresponding grouped evaluation. Classifier performance was summarized using the area under the receiver operating characteristic curve (AUROC) and average precision to account for class imbalance [39]. Because average precision depends on the positive-label prevalence, it was interpreted together with the observed rare-label definition and with prevalence-preserving permutation controls rather than as a prevalence-independent summary. As a negative control, rare stress labels were permuted independently within each task, preserving task size, feature distribution and class prevalence while disrupting the cell-level feature–label relationship. Thirty within-task permutations were evaluated using the same leave-task-out design and biology-plus-QC feature set.

### 2.6. External Validation Strategy

External validation analyses tested whether the same stress axes were recovered in independent stress-relevant contexts. GSE129757 bulk RNA sequencing (RNA-seq) data were used to test tunicamycin- and thapsigargin-induced UPR/ISR module activation in LN308 cells at the sample and gene-set levels. GSE251912 single-cell data were used to compare 24 h dimethyl sulfoxide (DMSO) and thapsigargin exposure across donors using cell-level, donor-paired and leave-donor-out analyses. GSE140819 non-small-cell lung cancer sample 14 (NSCLC14) protocol-stress data were used to evaluate collagenase 4 (C4), pronase/dispase/elastase/collagenase (PDEC) and Liberase/elastase (LE) dissociation protocols before and after adjustment for annotated cell type and QC covariates.

Unless otherwise stated, statistical tests were one-sided in the biologically specified direction of higher stress activity in the treated or higher-stress condition. Adjustments for multiple testing were made using the Benjamini–Hochberg false-discovery-rate (FDR) procedure [40]. Uncertainty for baseline-to-future prediction was estimated by non-parametric bootstrap confidence intervals over held-out cell-line–drug pairs [41]. Single-cell classifier confidence intervals were computed by resampling tasks rather than individual cells. In GSE251912, donor-clustered mixed models and binomial generalized estimating equation (GEE) analyses were used as supportive analyses for donor grouping, and in GSE140819, heteroskedasticity-robust linear models adjusted protocol effects for cell type and QC [42,43,44]. Core numerical calculations, statistical modeling and visualization were performed using Python packages 3.14.6 including NumPy (version 2.1.3), SciPy (version 1.14.1), scikit-learn (version 1.5.2), statsmodels (version 0.14.4), h5py (version 3.12.1), and Matplotlib (version 3.9.2) [37,45,46,47,48].

## 3. Results

### 3.1. Definition of Interpretable Stress-Program Axes and Rare High-Stress Populations

**Figure 1:**
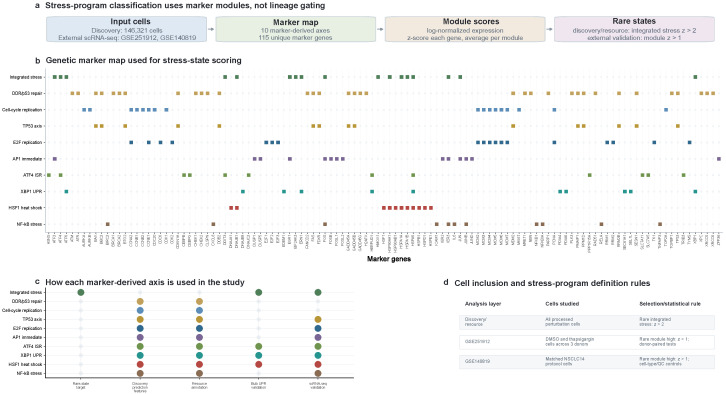
Definition of interpretable stress-program axes and rare high-stress populations. (**a**) Workflow from input cells through marker mapping and module scoring to rare-state definition. (**b**) Membership of 115 marker genes across 10 stress-program axes. (**c**) Use of each axis in prediction, resource annotation and external validation. (**d**) Cell-inclusion criteria and stress-state thresholds for the discovery and validation datasets. Colors distinguish workflow stages and stress-program axes; light gray marks indicate genes or axes not assigned to the corresponding category.

We first established an interpretable stress-program framework to define the stress axes and rare high-scoring populations used throughout the Results (Figure 1). Curated marker modules were organized into 10 partially overlapping regulatory axes, including integrated stress, *ATF4* ISR, *XBP1* UPR, *HSF1* heat shock, AP1 immediate response and replication-associated programs. These axes were treated as continuous module activities rather than mutually exclusive cell states. Rare high-stress populations were defined operationally as cells in the high-activity tail of integrated-stress or module-specific scores, depending on the analysis layer, so that “rare” denotes a thresholded stress-program outcome rather than a fixed cell proportion or lineage category. This definition provided a common measurement layer for the subsequent task-level prediction, single-cell classification and external validation analyses. The framework therefore allowed downstream analyses to ask whether pre-existing cellular heterogeneity and perturbation context predicted future stress-program emergence.

### 3.2. Baseline Cellular Heterogeneity Predicts Future Rare Integrated-Stress Burden

**Figure 2:**
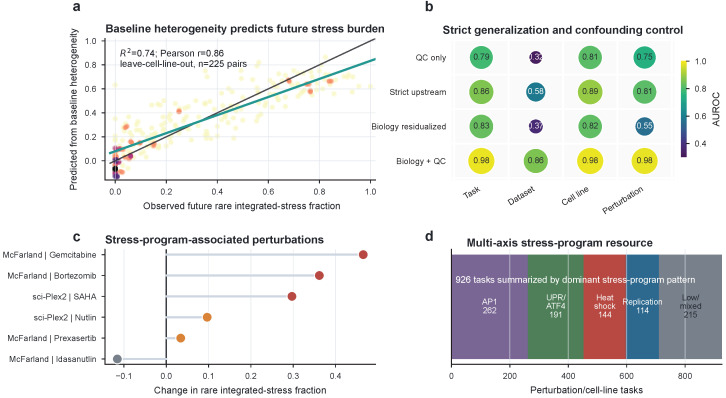
Baseline heterogeneity and perturbation context predict future rare integrated-stress burden and define a multi-axis resource. (**a**) Overview of the discovery framework spanning 146,321 single cells, 926 perturbation–cell-line tasks, four hold-out generalization designs and three independent validation datasets. The scatter plot depicts hold-out prediction of future rare integrated-stress burden from untreated baseline population features and drug indicators in the McFarland perturbation series. Each colored point represents one held-out cell-line–drug pair; the gray diagonal denotes the identity line, and the green line denotes the fitted calibration line. (**b**) Area under the receiver operating characteristic curve (AUROC) values for stress-program prediction across leave-task-out, leave-dataset-out, leave-cell-line-out and leave-perturbation-out splits and feature configurations. (**c**) Perturbation-level shifts in rare integrated-stress fraction relative to control. (**d**) Distribution of resource tasks across the dominant activator protein 1 (AP1) immediate, unfolded protein response (UPR)/activating transcription factor 4 (*ATF4*), heat shock, replication-coupled and low/mixed axes.

Using this framework, we next asked whether rare proteostasis-stress programs that emerge after perturbation could be anticipated from pre-existing cellular heterogeneity [6,9,19,22]. For the baseline-to-future analysis, we focused on the integrated-stress outcome and quantified the fraction of cells occupying the rare high-integrated-stress tail after perturbation. Across 146,321 single cells and 926 perturbation–cell-line tasks, this framework generated a stress-program resource spanning seven perturbation classes, 172 cell lines and two independent source compendia [3,4] (Figure 2).

Within this discovery resource, the operational rare fraction was variable rather than fixed by definition. Using the primary integrated_stress_z >2 threshold, rare high-integrated-stress fractions ranged from 0 to 1.0 across the 926 perturbation–cell-line tasks, with a median of 0.113 and an interquartile range of 0.032–0.267. This variability confirmed that rare-state burden was an observed task-level outcome rather than a fixed prevalence imposed during analysis, providing the quantitative endpoint for the baseline-to-future prediction test.

In the McFarland perturbation series, untreated population features together with drug indicators predicted the future fraction of rare high-integrated-stress cells across 225 held-out cell-line–drug pairs. In leave-cell-line-out cross-validation, the baseline-to-future model explained 74.2% of the variance in future rare integrated-stress burden ($R^{2}=0.742$, 95% bootstrap confidence interval (CI) 0.678–0.790; Pearson r=0.862, 95% CI 0.818–0.896; mean absolute error, 0.115, 95% CI 0.104–0.128; Figure 2a). Calibration was close to the identity line (slope, 0.984; 95% CI 0.900–1.059). These results indicate that future rare integrated-stress burden contains a predictable component associated with untreated population structure and perturbation identity, consistent with prior evidence that pre-existing transcriptional heterogeneity can be linked to later cellular outcomes [6,9,22].

Having established task-level predictability, we next moved to single-cell rare stress-program prediction and tested whether the signal generalized beyond the task on which a model was trained. Biology-plus-QC features yielded high performance under leave-task-out (AUROC, 0.980; average precision, 0.896), leave-cell-line-out (AUROC, 0.980; average precision, 0.896) and leave-perturbation-out (AUROC, 0.976; average precision, 0.872) splits and retained a predictive signal in the more stringent leave-dataset-out split (AUROC, 0.857; average precision, 0.698; Figure 2b). A strict upstream feature set that excluded direct UPR and heat-shock modules also generalized above QC-only baselines in most hold-out contexts, suggesting that rare integrated-stress prediction was linked to broader regulatory context instead of being captured only by final stress-marker activity.

The strongest perturbation-level shifts were not uniformly distributed across drugs, indicating that predictability was accompanied by substantial biological heterogeneity. Gemcitabine increased the rare integrated-stress fraction by 0.465 and Bortezomib by 0.362 in the McFarland compendium, while suberoylanilide hydroxamic acid (SAHA) produced a 0.297 increase in sci-Plex2; in contrast, idasanutlin decreased the rare integrated-stress fraction by 0.116 (Figure 2c). Classification of all resource tasks by their dominant stress-program patterns indicated a heterogeneous landscape comprising AP1 immediate (262 tasks), UPR/*ATF4* (191 tasks), heat shock (144 tasks), replication-coupled (114 tasks) and low/mixed (215 tasks) patterns (Figure 2d). Together, these results suggest that rare integrated-stress responses form a multi-axis landscape rather than a single generic stress continuum, motivating explicit control analyses and external validation of the inferred axes.

### 3.3. Rare Stress-Program Prediction Generalizes Across Hold-Out Contexts and Negative Controls

Because stress signatures in single-cell data can overlap with quality and processing effects, we next separated rare stress-program signal from technical or compositional confounding by benchmarking models across multiple feature sets and held-out designs. QC-only models were informative in some settings, consistent with partial coupling between stress-program variation and library-complexity features, but did not account for the full predictive signal. This control was motivated by prior observations that single-cell stress signatures can be influenced by dissociation, storage, enzymatic processing and low-quality-cell effects [17,18,23,49]. In the leave-dataset-out split, QC-only features did not generalize (AUROC, 0.323), whereas biology-plus-QC features reached an AUROC of 0.857 and an average precision of 0.698 (Figure 2b). Similarly, biology-residualized features retained predictive signal in leave-task-out and leave-cell-line-out tests (AUROC, 0.828 and 0.818, respectively), indicating that rare integrated-stress prediction was not reducible to raw count depth or gene complexity.

**Figure 3:**
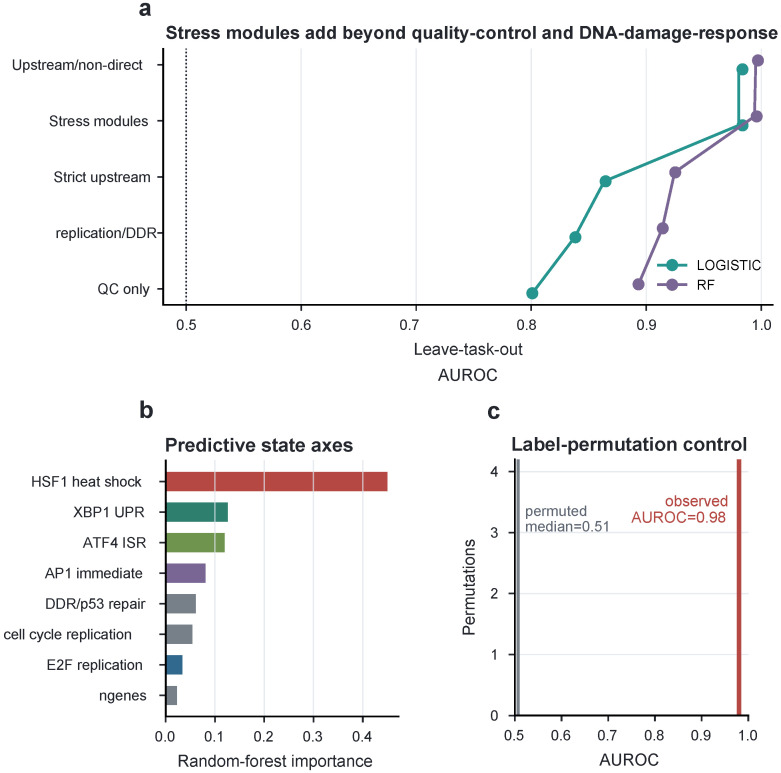
Rare stress-program prediction generalizes across hold-out contexts and negative controls. (**a**) Leave-task-out area under the receiver operating characteristic curve (AUROC) for logistic-regression and random-forest models across quality-control (QC), replication/DNA damage response (DDR), stress-module and upstream/non-direct feature sets. The dotted vertical line indicates chance-level performance (AUROC = 0.5). (**b**) Random-forest feature importance for the upstream/non-direct stress feature set. (**c**) Within-task label-permutation negative control compared with the observed biology-plus-QC model.

Model comparisons were consistent with this pattern. In leave-task-out prediction, logistic models using stress-module features yielded an AUROC of 0.980 and an average precision of 0.895, compared with an AUROC of 0.801 and average precision of 0.553 for QC-only features. Random-forest models yielded higher absolute performance while preserving the same ordering: stress-module and upstream/non-direct feature sets reached AUROC values of 0.994–0.995, whereas QC-only features reached an AUROC of 0.893 (Figure 3a). Task-clustered bootstrap intervals were consistent with high random-forest stress-module performance after resampling tasks rather than cells (AUROC, 0.994; 95% CI 0.990–0.997; average precision, 0.971; 95% CI 0.956–0.979). Feature-importance analysis ranked the *HSF1* heat shock, *XBP1* UPR, *ATF4* ISR and AP1 immediate axes among the dominant predictive components, with additional contributions from DDR and cell-cycle features [36,41] (Figure 3b).

As a complementary negative control, we performed within-task label permutation. Rare-stress labels were permuted within each task, preserving task size, class prevalence and feature distributions while disrupting the cell-level association between module features and rare integrated-stress status. This procedure reduced performance to near-null values (median AUROC, 0.507; 95% permutation interval 0.496–0.514; median average precision, 0.154), whereas the observed biology-plus-QC model reached an AUROC 0.980 in the matched leave-task-out setting (empirical permutation p=0.032 for both AUROC and average precision; Figure 3c). Together, these analyses indicate that rare integrated-stress prediction is unlikely to be explained by QC covariates, task structure or class imbalance alone, although QC-associated variation remains an important component to control. Thus, the classifier was interpreted as detecting cell-level stress-axis configuration within the scored feature space, instead of merely separating globally strong from weak perturbation contexts.

### 3.4. Canonical Endoplasmic Reticulum (ER) Stress Perturbations Activate the Inferred UPR/ISR Modules

Having established internal robustness, we next sought external support in a perturbation setting directly aligned with ER stress and UPR/ISR biology. GSE129757 profiles LN308 glioblastoma cells at 2 and 6 h after tunicamycin or thapsigargin treatment, providing an independent bulk RNA-seq dataset focused on canonical ER stress activation [24]. Marker coverage was complete or nearly complete for the stress modules, enabling direct testing of module-level enrichment.

**Figure 4:**
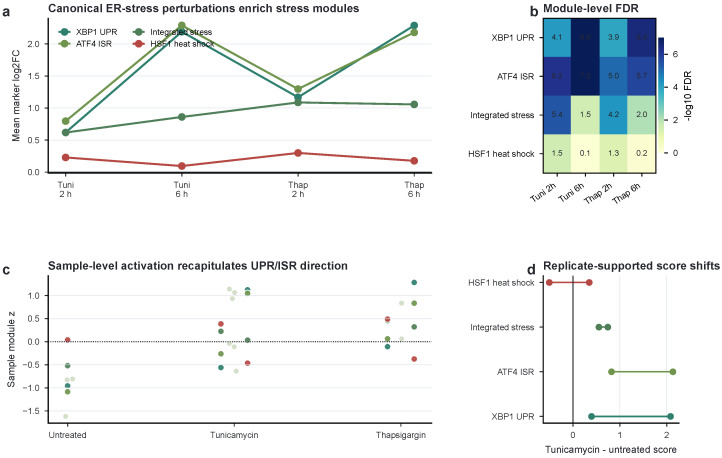
Canonical endoplasmic reticulum (ER) stress perturbations activate inferred unfolded protein response/integrated stress response (UPR/ISR) modules in an external bulk RNA sequencing (RNA-seq) dataset. (**a**) Mean marker log_2_ fold change for stress modules after tunicamycin or thapsigargin treatment in GSE129757. (**b**) Module-level differential-expression enrichment quantified as $-\log_{10}$ false-discovery rate (FDR), with the heatmap color scale representing $-\log_{10}$ FDR. (**c**) Sample-level module scores across untreated, tunicamycin-treated and thapsigargin-treated samples. (**d**) Replicate-supported tunicamycin score shifts relative to untreated controls. Colors consistently distinguish the integrated-stress, *HSF1* heat-shock, *XBP1* UPR and *ATF4* ISR modules across panels.

Both perturbagens were consistent with activation of the expected UPR/ISR axes. In gene-level differential-expression contrasts, the *ATF4* ISR module was strongly enriched after at 6 h tunicamycin (mean marker log_2_ fold change, 2.290; FDR, $9.61\times 10^{-8}$) and at 6 h after thapsigargin (mean marker log_2_ fold change, 2.179; FDR, $1.97\times 10^{-6}$). The *XBP1* UPR module was similarly enriched after at 6 h tunicamycin (mean marker log_2_ fold change, 2.191; FDR, $1.12\times 10^{-7}$) and at 6 h after thapsigargin (mean marker log_2_ fold change, 2.288; FDR, $3.75\times 10^{-7}$; Figure 4a,b), consistent with canonical ER stress activation through *ATF4* ISR and *XBP1* UPR pathways [13,27,28,29,50]. Integrated-stress markers were also enriched across tunicamycin and thapsigargin contrasts, whereas the heat shock module was not the dominant response in this ER stress context.

Sample-level module scores were directionally concordant with the gene-level enrichment results. Tunicamycin increased *XBP1* UPR and *ATF4* ISR scores at 6 h, and thapsigargin produced the largest descriptive shifts in *XBP1* UPR and *ATF4* ISR at 6 h (Figure 4c,d). Because some thapsigargin time points contained only one treated sample, sample-level score comparisons for those time points were interpreted descriptively, and statistical support was based primarily on the supplied gene-level differential-expression contrasts. Thus, GSE129757 provided external gene-level evidence consistent with activation of the UPR/ISR modules used in the stress-program framework by canonical ER stress perturbations.

### 3.5. Direct Single-Cell Thapsigargin Perturbation Is Consistent with High-UPR/ISR State Expansion

Because bulk RNA-seq cannot resolve whether pathway activation reflects broad low-level shifts or expansion of high-scoring cells, we then tested the stress-program framework in an independent single-cell ER stress perturbation dataset. GSE251912 contains human islet single-cell RNA-seq after 24 h DMSO or thapsigargin exposure across three donors, allowing both cell-level and donor-paired assessment. After processing, the analysis included 55,577 cells, comprising 29,063 thapsigargin-treated and 26,514 DMSO control cells. This design allowed us to ask whether the UPR/ISR modules validated in bulk data were also associated with rare high-state expansion at single-cell resolution.

**Figure 5:**
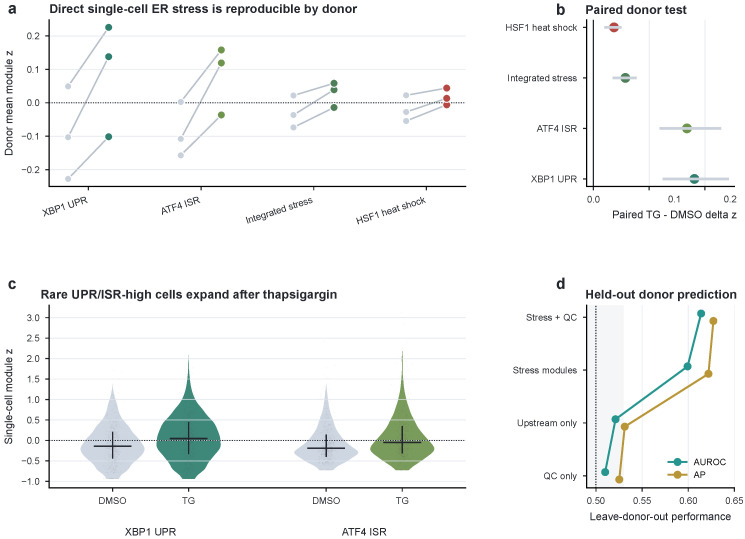
Direct single-cell thapsigargin perturbation is consistent with high-unfolded protein response/integrated stress response (UPR/ISR) state expansion. (**a**) Donor-paired module scores after 24 h dimethyl sulfoxide (DMSO) or thapsigargin treatment; gray and colored points denote DMSO and thapsigargin, and lines link the same donors. (**b**) Paired thapsigargin–DMSO score deltas; points show means and gray bars show 95% confidence intervals. (**c**) Single-cell X-box binding protein 1 (*XBP1*) UPR and activating transcription factor 4 (*ATF4*) ISR distributions; gray and colored violins denote DMSO and thapsigargin, and black bars show the mean and standard deviation. (**d**) Leave-donor-out prediction across feature sets; green and ochre denote the area under the receiver operating characteristic curve (AUROC) and average precision (AP). The dotted line marks AUROC = 0.5, and the gray area spans the chance-reference values.

Thapsigargin was associated with increased UPR/ISR module activity across donors (Figure 5a). In donor-paired tests, *XBP1* UPR increased by a mean paired delta of 0.181 z units (95% CI 0.038–0.324; FDR, 0.0349), *ATF4* ISR by 0.168 z units (95% CI 0.033–0.302; FDR, 0.0349), integrated stress by 0.057 z units (95% CI 0.0088–0.106; FDR, 0.0349) and *HSF1* heat shock by 0.037 z units (95% CI 0.0026–0.071; FDR, 0.0349; Figure 5b). Because the paired analysis included three donors, donor-level tests were interpreted as supportive and were considered together with cell-level effect sizes and donor-clustered models.

At the single-cell level, *XBP1* UPR had the largest shift after thapsigargin exposure (delta mean z, 0.181; Hedges’ g=0.348; Cliff’s delta, 0.192; high-module z>1 fraction increase, 0.0358; FDR $<10^{-300}$), followed by *ATF4* ISR (delta mean z, 0.165; Hedges’ g=0.323; Cliff’s delta, 0.179; high-module z>1 fraction increase, 0.0373; FDR, $2.98\times 10^{-292}$) and integrated stress (delta mean z, 0.0537; FDR, $1.98\times 10^{-70}$; Figure 5c). Donor-clustered GEE models were consistent with increased odds of high-*XBP1* UPR cells (odds ratio, 2.72; 95% CI 1.92–3.84) and high-*ATF4* ISR cells (odds ratio, 2.36; 95% CI 1.81–3.08). Donor-paired rare-state fractions varied across donors and shifted upward after thapsigargin: *XBP1* UPR z>1 fractions ranged from 0.0117 to 0.0344 after DMSO and 0.0326 to 0.0722 after thapsigargin, whereas *ATF4* ISR z>1 fractions ranged from 0.0209 to 0.0382 after DMSO and 0.0460 to 0.0857 after thapsigargin.

Finally, we asked whether these stress-program features generalized across donors in treatment prediction. In leave-donor-out models, stress modules alone outperformed QC-only features (AUROC, 0.599 versus 0.510; average precision, 0.622 versus 0.525), and adding QC features modestly improved performance (AUROC, 0.614; average precision, 0.627; Figure 5d). In contrast, an upstream-only feature set that excluded the direct UPR axes performed near the QC-only baseline (AUROC, 0.521). This direct single-cell ER stress perturbation dataset was therefore consistent with the expected direction of UPR/ISR activation and with expansion of high-*XBP1* UPR and high-*ATF4* ISR cells after thapsigargin exposure.

### 3.6. An Independent Single-Cell Protocol-Stress Dataset Indicates Heat Shock/Proteostasis Structure Beyond Cell Type and QC

To determine whether external support extended beyond pharmacological ER stress, we next evaluated whether the stress-program modules detected single-cell proteostasis stress in an independent NSCLC14 dissociation-protocol dataset from GSE140819. This dataset provides matched single-cell profiles generated under cold-active protease, protease with DNase/elastase/collagenase, and Liberase-based protocols, a setting associated with protocol-dependent stress responses. The filtered analysis included 13,866 cells with cell-type annotations and QC metrics.

**Figure 6:**
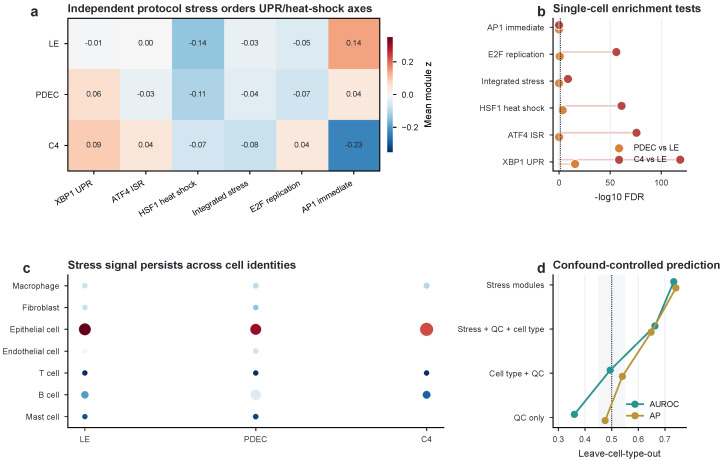
Independent single-cell protocol-stress validation indicates heat shock/proteostasis structure beyond cell type and quality control (QC). (**a**) Mean module scores across Liberase/elastase (LE), pronase/dispase/elastase/collagenase (PDEC) and collagenase 4 (C4) dissociation protocols in the non-small-cell lung cancer sample 14 (NSCLC14) subset of GSE140819. (**b**) One-sided single-cell enrichment tests for PDEC and C4 relative to LE; horizontal segments extend from zero to the corresponding $-\log_{10}$ FDR values. (**c**) Protocol-associated stress scores across major annotated cell types. (**d**) Leave-cell-type-out prediction of C4 versus LE using QC, cell-type and stress-module feature sets. Green and ochre lines indicate AUROC and average precision, respectively; the dotted line marks AUROC = 0.5, and the gray band indicates chance-reference values. Stress-axis abbreviations include X-box binding protein 1 (*XBP1*)-associated unfolded protein response (UPR), heat shock factor 1 (*HSF1*)-associated heat shock response and activator protein 1 (AP1)-mediated immediate-response programs.

Relative to the Liberase reference, the warm-collagenase C4 protocol increased *XBP1* UPR (delta mean z, 0.227; FDR, $3.19\times 10^{-119}$), *ATF4* ISR (delta mean z, 0.178; FDR, $1.82\times 10^{-76}$), *HSF1* heat-shock (delta mean z, 0.178; FDR, $7.70\times 10^{-62}$) and integrated stress (delta mean z, 0.0605; FDR, $1.84\times 10^{-9}$; Figure 6a,b). PDEC had a weaker but significant increase in *XBP1* UPR (delta mean z, 0.0813; FDR, $2.09\times 10^{-16}$) and *HSF1* heat shock (delta mean z, 0.0349; FDR, $2.55\times 10^{-4}$), consistent with a graded protocol-associated stress pattern. After adjustment for annotated cell type and QC covariates using heteroskedasticity-robust linear models, *HSF1* heat shock had the most stable positive protocol-associated signal for C4 versus LE (adjusted delta z, 0.0977; 95% CI 0.0807–0.115; FDR, $6.76\times 10^{-29}$), whereas several UPR and integrated-stress differences were attenuated or reversed, suggesting that these components were partly coupled to cell composition or QC.

The protocol-associated signal was not confined to a single annotated cell type. Across major cell classes, stress-module scores remained detectable after stratification by cell identity, although the magnitude varied by lineage (Figure 6c). Consistent with this lineage dependence, high-module fractions varied across protocol–cell-type strata rather than taking a fixed value across cell classes. In leave-cell-type-out prediction of C4 versus Liberase, stress modules alone yielded an AUROC of 0.733 and an average precision of 0.741, exceeding QC-only features (AUROC, 0.360; average precision, 0.476) and cell-type-plus-QC features (AUROC, 0.495; average precision, 0.540; Figure 6d). These results indicate that the stress-program framework can help detect protocol-associated heat shock/proteostasis structure while suggesting that UPR and integrated-stress components in dissociation datasets require careful interpretation with respect to cell composition and QC.

### 3.7. A Cross-Dataset Stress-Program Resource Stratifies Perturbation-Specific Proteostasis Programs

**Figure 7:**
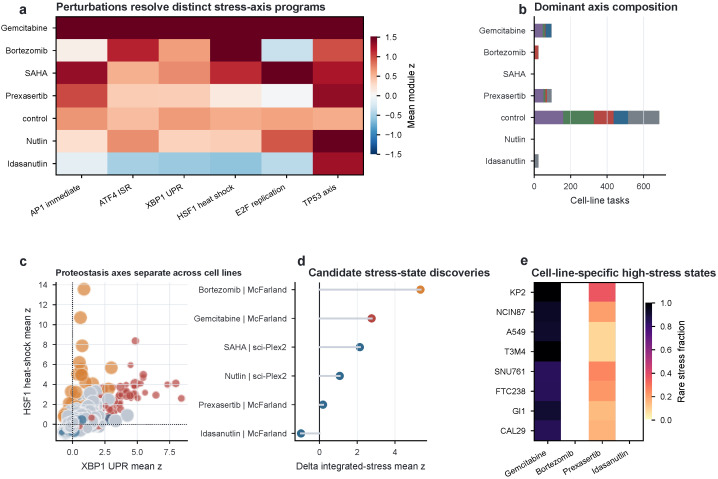
A cross-dataset resource identifies perturbation- and cell-line-specific stress programs. (**a**) Perturbation-level stress-program profiles averaged across resource tasks; color intensity indicates the mean module *z*-score. (**b**) Dominant stress-program pattern composition by perturbation, with colors distinguishing stress-program patterns. (**c**) Cell-line-level separation of X-box binding protein 1 (*XBP1*) unfolded protein response (UPR) and heat shock factor 1 (*HSF1*) heat shock axes across perturbations; colors distinguish stress-program patterns. (**d**) Top perturbation-level increases in integrated-stress mean score, colored by stress-program pattern. (**e**) Cell-line-specific rare integrated-stress fractions across selected perturbations; color intensity indicates the rare stress fraction.

Finally, we returned to the full stress-program resource to ask whether the predictable stress burden resolved into qualitatively distinct perturbation-specific programs. Averaging module scores by perturbation suggested separable profiles: gemcitabine and bortezomib produced high rare integrated-stress burdens but differed in the balance of AP1 immediate, UPR, heat shock, replication and *TP53*-related axes; prexasertib had a weaker and more heterogeneous stress profile; and idasanutlin produced low rare integrated-stress fractions despite *TP53*-axis engagement (Figure 7a,b). Across all tasks, gemcitabine had the largest mean rare integrated-stress fraction (0.566), followed by bortezomib (0.483) and SAHA (0.312), whereas idasanutlin had the smallest fraction (0.012). Median rare fractions showed the same perturbation dependence, with gemcitabine tasks at 0.625, bortezomib at 0.494, prexasertib at 0.100 and idasanutlin at 0.0047.

At the cell-line level, the same perturbation could resolve into different dominant stress-program patterns. For example, gemcitabine-treated cell lines included AP1 immediate, heat shock and replication-coupled high-stress tasks, with rare integrated-stress fractions approaching 1.0 in several cell lines (Figure 7c–e). The top resource entries included gemcitabine-treated T3M4 and KP2 cells, each with a rare integrated-stress fraction of 1.0 and AP1 immediate dominance, whereas gemcitabine-treated PATU8988T cells had a high rare integrated-stress fraction with heat-shock dominance. Across resource tasks, rare integrated-stress burden was most strongly associated with *HSF1* heat-shock mean score (Spearman ρ=0.847, FDR $=2.54\times 10^{-255}$), followed by the *ATF4* ISR, AP1 immediate, *XBP1* UPR and E2F replication axes. Task rankings were stable across rare high-stress threshold choices: rare fractions defined at z>1.0, z>1.5 and z>2.5 correlated strongly with the primary z>2.0 definition (Spearman ρ=0.937, 0.971 and 0.974, respectively). These examples indicate that the resource stratifies perturbation–cell-line tasks by both rare integrated-stress burden and dominant stress-program pattern structure instead of merely ranking perturbations by a single stress score. Accordingly, rare integrated-stress burden should be interpreted as an operational summary of the high-scoring tail of a continuous module-score distribution, whereas dominant stress-program pattern labels describe relative program balance rather than discrete, mutually exclusive biological categories.

Together, these analyses define a cross-dataset stress-program prediction resource in which baseline cellular heterogeneity and perturbation context anticipate rare integrated-stress emergence; predictive signals persist under stringent hold-out and negative-control tests; and independent ER stress, thapsigargin and protocol-stress datasets provide external support for the inferred stress axes. This resource provides a hypothesis-generating map of context-specific proteostasis programs across perturbation–cell-line settings.

## 4. Discussion

Stress-associated transcriptional programs are often treated as inconvenient variation in single-cell perturbation data because they can reflect biology, experimental handling, cell damage or technical quality differences [23,49]. Here, our analyses suggest that rare proteostasis-stress programs are unlikely to be solely nuisance signals. Across public single-cell perturbation data, baseline cellular heterogeneity and perturbation identity predicted future rare integrated-stress burden; single-cell stress-program prediction generalized across held-out tasks, cell lines, perturbations and datasets; and independent UPR, thapsigargin and protocol-stress datasets were consistent with the inferred axes. These results are consistent with a view in which stress responses constitute an organized and partly predictable dimension of cellular response space.

A central implication is that rare high-stress populations should not automatically be interpreted as stochastic outliers. Untreated population features, interpreted together with drug identity, contained information about which cell-line–drug pairs later generated rare high-integrated-stress populations. This interpretation is consistent with prior evidence that rare or pre-existing transcriptional states, drug-tolerant trajectories and state-to-fate relationships can be associated with later treatment responses or cellular outcomes [8,9,10,11,19,22]. This does not imply that future stress fate is predetermined at the single-cell level, nor that the measured baseline features are causal drivers. Rather, it suggests that pre-existing regulatory heterogeneity creates a permissive context in which entry into the rare high-stress category becomes more or less likely after a specific perturbation. In this usage, a permissive context is not a single *HSF1*- or *XBP1*-defined factor but a broader cellular-context configuration that may alter the probability or amplitude of stress-program entry. The source of this baseline heterogeneity is likely multifactorial: it may include differences in transcriptional regulatory activity, metabolic state, basal proteostasis capacity, replication state, immediate early signaling, inflammatory or damage-associated activity, and chromatin accessibility or chromatin-associated drug-tolerant states described in other systems [7,51,52]. Because the discovery compendium does not directly measure chromatin accessibility, protein abundance or metabolic flux, the baseline predictors cannot distinguish whether high-stress susceptibility is driven by metabolic state, epigenetic state, proteostasis-network capacity or a combination of those factors. Although perturbation intensity probably contributes to stress induction, the persistence of predictive signal in upstream-feature models, held-out perturbation settings and perturbation–cell-line-specific axis profiles suggests that the framework captures biological context beyond a simple distinction between strong and weak perturbation.

The resource is also consistent with a multi-axis stress landscape rather than a single generic stress continuum. Perturbation–cell-line tasks separated into AP1 immediate, UPR/*ATF4*, heat shock, replication-coupled and low/mixed dominant stress-program patterns. This distinction is biologically important because these programs imply different mechanisms: *HSF1*-dominant patterns are consistent with heat-shock regulation and inducible chaperone networks [30,31], UPR/*ATF4*-dominant patterns are consistent with ER stress and integrated-stress signaling [13,29,50], AP1-dominant patterns may reflect acute immediate-early adaptation [32], and replication-coupled patterns may indicate interaction with G1/S transcriptional programs [33]. Accordingly, *HSF1* heat shock and *XBP1* UPR axes should be interpreted as stress-response dimensions embedded within a broader cellular context rather than as isolated pathways. The models and validations indicate that these axes are reproducible and informative, but they do not imply independence from metabolic, epigenetic or cell-cycle context.

Independent validation reduced overfitting concerns. In the bulk ER stress dataset, GSE129757 indicated tunicamycin- and thapsigargin-induced UPR/ISR activation at the gene level [24]. In the thapsigargin single-cell dataset, GSE251912 indicated donor-paired expansion of *XBP1* UPR and *ATF4* ISR activity [25]. GSE140819 addressed a distinct but important question by testing whether an independent single-cell protocol-stress dataset contains heat shock/proteostasis structure beyond cell type and QC. The persistence of heat shock signal after these adjustments is consistent with the interpretation that protocol-associated *HSF1*/proteostasis variation is unlikely to be simply a low-quality-cell artifact. Instead, it may represent a biological response to sample handling that can interact with cell identity and should be annotated rather than automatically removed in atlas or perturbation analyses [26].

This study has several limitations. First, the analyses are computational and observational; therefore, they support predictability and structured association but do not establish that a specific baseline module, cellular condition or perturbation mechanism causally drives entry into rare high-stress states. Second, module scores are intentionally interpretable but coarse, and they do not model full regulatory networks, post-transcriptional control or protein-level stress signaling. Third, the definition of a rare high-stress population depends on an operational z-score cutoff. We used integrated_stress_z >2.0 as the primary discovery threshold and evaluated sensitivity across z>1.0 to z>2.5, but threshold-based definitions should be interpreted as analytical conventions rather than discrete biological boundaries. Fourth, dataset heterogeneity remains an important constraint: the discovery and validation datasets differ in cell type, perturbation class, exposure time, assay design, sample handling and processing pipeline, which strengthens generalizability but limits direct quantitative comparability. Finally, some adjusted protocol-stress analyses suggested attenuation of UPR and integrated-stress effects after accounting for cell type and QC, indicating that these axes may be partly coupled to composition or technical covariates [23,49].

Despite these limitations, the framework provides a practical way to use stress variation rather than simply remove it. It may help identify perturbations that selectively produce rare populations with high activity of the UPR, ISR, heat shock, or AP1 programs; distinguish protocol-associated stress from lineage-associated biology; and nominate cell-line-specific proteostasis vulnerabilities that may be missed by population averages. This application is consistent with single-cell drug-response studies showing that rare or heterogeneous transcriptional programs can accompany drug tolerance and context-specific vulnerabilities, as well as work establishing UPR/ISR and proteostasis pathways as stress-adaptation systems [4,10,11,12,13,14]. These features make stress-program stratification a potential complement to conventional drug-response prediction and perturbation design and may inform future strategies for selecting or designing perturbation contexts rather than serve as an immediate basis for clinical treatment choice. Future studies could ask whether a perturbation selectively expands rare high-stress subpopulations; use baseline stress-program scores to stratify cell lines or dosing schedules; and combine time-resolved perturbation, single-cell clustered regularly interspaced short palindromic repeats (CRISPR) approaches [53], chromatin, metabolic and protein-level measurements to test whether baseline predictors are causes, correlates or consequences of broader cellular context. For example, baseline profiling could be used in future prospective studies to flag contexts in which drugs such as gemcitabine or bortezomib are expected to drive broad high-stress responses or to identify idasanutlin-like contexts in which *TP53*-axis engagement occurs without large rare integrated-stress expansion. Such information could help prioritize perturbations that avoid, exploit or experimentally probe rare high-stress populations. In summary, rare proteostasis-stress programs appear to be partly predictable outcomes associated with cellular heterogeneity and perturbation context, rather than only technical artifacts or terminal consequences of perturbation. By integrating baseline prediction, single-cell stress-program classification, confounding controls and independent UPR/heat-shock validation datasets, this work helps reframe stress responses as a structured biological layer of perturbation biology.

## 5. Conclusions

This study suggests that rare proteostasis-stress programs are partly predictable, structured and consistent with independent validation datasets. Rather than treat stress signatures only as confounders to remove, the framework developed here uses them to map how baseline heterogeneity, perturbation identity and cellular context shape rare stress-program emergence. The resulting resource distinguishes mechanistically different stress axes and identifies perturbation- and cell-line-specific proteostasis programs that may be missed by population-average analyses.

The findings do not imply that baseline profiles directly cause future stress fate; prospective perturbation and functional experiments would be needed to test causality. Nonetheless, the combination of baseline features, perturbation context, stringent hold-out evaluation, negative controls and independent validation is consistent with a practical shift in how stress variation is interpreted. Rare populations with high activity of the UPR, ISR, heat shock, or AP1 programs, as well as replication-coupled stress-program populations, may be treated as analyzable biological outcomes, and the resource of 146,321 single cells across 926 perturbation–cell-line tasks may provide a reference for future benchmarking and stress-program model refinement.

## Data Availability

The public datasets analyzed in this study are available from the Gene Expression Omnibus under accession numbers GSE129757, GSE140819 and GSE251912 and from the published single-cell perturbation resources cited in the manuscript. Processed result tables supporting the analyses are provided as Supplementary Materials. No new raw sequencing data were generated. The custom analysis scripts are not currently deposited in a public repository but can be made available by the corresponding author upon reasonable request.

## Supplementary Materials

The following supporting information can be downloaded at: https://www.mdpi.com/article/10.3390/biology15151230/s1: Supplementary Methods; processed result tables; model-performance summaries; permutation and threshold-sensitivity analyses; external validation statistics for GSE129757, GSE140819 and GSE251912; and selected perturbation–cell-line stress-program summary tables supporting the analyses.
