# Supplementary material for: Pre-Existing Heterogeneity Predicts Rare Proteostasis-Stress Programs Across Diverse Perturbations
Source: Biology (Basel). 2026 Jul 23;15(15):1230. doi: 10.3390/biology15151230 (PMC13464853; doi:10.3390/biology15151230)
Supplement: Supplementary file 1 [file biology-15-01230-s001.zip › Supplementary Materials.pdf]

Supplementary Materials

Pre-Existing Heterogeneity Predicts Rare Proteostasis-Stress Programs Across Diverse Perturbations

Zongnan Lyu, Chunxue Shao, Renyu Yang, Qi Yu, Guang Yang, and Ziheng Wang

Supplementary Methods

GSE129757 bulk RNA sequencing (RNA-seq) processing

For GSE129757, RNA-seq count files corresponding to LN308 samples were parsed from Gene Expression Omnibus (GEO)-supplied `GSM*_LN308-*.txt.gz` files. Ribosome profiling (Ribo-seq) and barcode-labelled files were excluded. Sample metadata were parsed from file names to identify untreated controls and tunicamycin- or thapsigargin-treated samples at 2 and 6 hours. Ensembl gene identifiers were stripped of version suffixes and mapped to gene symbols using the GEO-supplied processed differential-expression workbook. Counts were aggregated by gene symbol, first expressed as counts per million (CPM) and then converted to $\log_2(\text{CPM} + 1)$, gene-wise z-scored across samples and averaged across detected module genes.

Sample-level treatment effects were summarized as treated minus untreated mean module-score differences. When both treated and untreated groups contained at least two samples, treated samples were compared with untreated RNA-seq replicates using Welch two-sample t-tests; otherwise, the comparison was reported descriptively. Gene-level differential-expression enrichment used the GEO-supplied processed differential-expression table. For each contrast and module, module-gene $\log_2$ fold changes were compared with the protein-coding background using one-sided Mann–Whitney U tests, with false-discovery rates computed across module–contrast tests.

GSE251912 single-cell thapsigargin processing

For GSE251912, demultiplexed Matrix Market files were analyzed for donors 150, 162 and 168 under 24 h dimethyl sulfoxide (DMSO) and thapsigargin conditions. Gene-symbol, barcode and matrix files were read for each donor–condition sample. If matrix dimensions did not match the gene-list orientation, the matrix was transposed. Per-cell total counts and detected-gene counts were calculated from the full matrix. Marker-gene count matrices were then extracted for all genes present in the stress modules and concatenated across samples.

Module scores were computed after normalization to 10,000 counts per cell, $\log(1+x)$ transformation, gene-wise z-scoring across cells and averaging of detected module genes. Cells were labelled as treated if they came from the thapsigargin condition. Module-high cells in this external validation layer were summarized using the more permissive $z > 1$ threshold. Cell-level shifts were tested using one-sided Mann–Whitney U tests, donor-paired mean-score shifts were computed as thapsigargin

minus DMSO donor means, and leave-donor-out logistic regression used donor as the grouping variable.

GSE140819 single-cell protocol-stress processing

For GSE140819, the non-small-cell lung cancer sample 14 (NSCLC14) fresh collagenase 4 (C4), pronase/dispase/elastase/collagenase (PDEC) and Liberase/elastase (LE) samples were analyzed. Metadata files were filtered to remove cells annotated as empty droplets or doublets. Cell barcodes were matched to the corresponding gzipped 10x Hierarchical Data Format 5 (HDF5) matrices. To keep the validation targeted to the stress-program hypothesis, only genes belonging to the stress modules were extracted. Marker counts from all samples were concatenated, normalized to 10,000 counts per cell, transformed with $\log(1 + x)$, gene-wise z-scored and averaged to produce module scores.

Protocol summaries were computed by protocol and annotated cell type. LE was used as the lower-stress reference protocol. C4 versus LE and PDEC versus LE were compared using one-sided Mann–Whitney U tests. Confounding-control models for C4 versus LE used quality-control (QC)-only variables, cell-type-plus-QC variables, stress modules alone, and stress modules plus QC and cell-type code. Standardized logistic regression with balanced class weights was evaluated using `GroupKFold` cross-validation grouped by annotated cell type.

Software implementation

Analyses were implemented in Python using NumPy, pandas, SciPy, scikit-learn, statsmodels, h5py and Matplotlib. The same module definitions, normalization principles and multiple-testing correction strategy described in the main Methods were applied across discovery and validation analyses unless otherwise specified.
